# Prediction of incidence of neurological disorders in HIV-infected persons in Taiwan: a nested case–control study

**DOI:** 10.1186/s12879-023-08761-4

**Published:** 2023-11-04

**Authors:** Ya-Wei Weng, Susan Shin-Jung Lee, Hung-Chin Tsai, Chih-Hui Hsu, Sheng-Hsiang Lin

**Affiliations:** 1 Institute of Clinical Medicine, College of Medicine, National Cheng Kung University, Tainan, Taiwan; 2 Division of Infectious Disease, Department of Internal Medicine, Kaohsiung Veterans General Hospital, Kaohsiung, Taiwan; 3 Faculty of Medicine, School of Medicine, National Yang Ming Chiao Tung University, Taipei, Taiwan; 4 School of Medicine, College of Medicine, National Sun Yat-Sen University, Kaohsiung, Taiwan; 5 Institute of Biomedical Sciences, National Sun Yat-Sen University, Kaohsiung, Taiwan; 6 Biostatistics Consulting Center, National Cheng Kung University Hospital, College of Medicine, National Cheng Kung University, Tainan, Taiwan; 7 Department of Public Health, College of Medicine, National Cheng Kung University, Tainan, Taiwan

**Keywords:** HIV, Neurological disorders, National health insurance research database

## Abstract

**Background:**

Neurological disorders are still prevalent in HIV-infected people. We aimed to determine the prevalence of neurological disorders and identify their risk factors in HIV-infected persons in Taiwan.

**Methods:**

We identified 30,101 HIV-infected people between 2002 and 2016 from the National Health Insurance Research Database in Taiwan, and analyzed the incidence of neurological disorders. We applied a retrospective, nested case–control study design. The individuals with (case group) and without (control group) a neurological disorder were then matched by age, sex and time. Factors associated with neurological disorders were analyzed using a conditional logistic regression model, and a nomogram was generated to estimate the risk of developing a neurological disorder.

**Results:**

The incidence of neurological disorders was 13.67 per 1000 person-years. The incidence remained stable during the observation period despite the use of early treatment and more tolerable modern anti-retroviral therapy. The conditional logistic regression model identified nine clinical factors and comorbidities that were associated with neurological disorders, namely age, substance use, traumatic brain injury, psychiatric illness, HIV-associated opportunistic infections, frequency of emergency department visits, cART adherence, urbanization, and monthly income. These factors were used to establish the nomogram.

**Conclusion:**

Neurological disorders are still prevalent in HIV-infected people in Taiwan. To efficiently identify those at risk, we established a nomogram with nine risk factors. This nomogram could prompt clinicians to initiate further evaluations and management of neurological disorders in this population.

**Supplementary Information:**

The online version contains supplementary material available at 10.1186/s12879-023-08761-4.

## Background

Due to the widespread use of combination antiretroviral therapy (cART), the life expectancy of individuals infected with human immunodeficiency virus (HIV) has improved and even approaches that of the general population [1]. However, a gap remains in comorbidity-free years between HIV-infected individuals and the general population [2]. In addition to comorbidities including cardiovascular diseases, cancers, diabetes, dyslipidemia and chronic renal diseases, which are prevalent in people living with HIV (PLWH)[3, 4], neuropsychiatric conditions are also common in PLWH [5]. The neurological complications of HIV are quite diverse, and in the early stages of infection can include meningitis, encephalitis and Bell's palsy. Late-stage symptoms include HIV-associated neurocognitive disorders, toxoplasma encephalitis, tuberculous meningitis, cryptococcal meningitis and neurosyphilis [6]. As with the other HIV-associated comorbidities, HIV-associated neurocognitive disorders are still prevalent in the modern cART era, with an overall prevalence rate of around 45% [7, 8]. These disorders can affect the quality of life and contribute to mortality in PLWH [9]. The pattern of HIV-associated neurocognitive disorders has changed in the recent two decades [10], and the prevalence may be underestimated due to a lack of awareness [11].

HIV also affects the central nervous system early in infection [12], and blood–brain barrier disruption has been demonstrated early in the course of primary HIV infection [13]. Thus, central nervous system infection caused by primary HIV infection or other pathogens (virus, bacteria, fungi) is also a common neurological complication in HIV-infected patients. However, there are limited data about neurological disorders in PLWH in the Asia–Pacific region [14, 15].

In Taiwan, cART has been provided free of charge since 1997. However, guidelines for the diagnosis and treatment of HIV/AIDS in Taiwan have recommended initiating cART according to different CD4 cell levels at different times: < 200 cells/mm^3^ in 2006, < 350 cells/mm^3^ in 2010, < 500 cells/mm^3^ in 2013, and "treat all" since 2016. Improvement in treatment coverage for PLWH was also implemented in other countries due to new scientific evidence around HIV treatment during this period of time [16]. Several studies have reported that CD4 nadir and CD4 count are predictors of HIV neurological disorders in the era of modern cART [17–19]. Thus, there may have been dynamic changes or even improvements in neurological disorders in PLWH in Taiwan during this time.

Several clinical factors and comorbidities have been reported to contribute to cognitive impairment in PLWH, including advanced HIV disease [17], duration of HIV infection [20, 21], obesity and diabetes [22], increased age [23], and hepatitis C infection [23]. In addition, alcohol use, substance abuse, traumatic brain injury, sleep disorders and psychiatric illnesses may also predispose to cognitive disorders in PLWH [24].

In the present study, we aimed to determine the dynamic changes in neurological disorders from 2002 to 2017, and to identify risk factors for neurological disorders in HIV-infected persons even under different treatment strategies in Taiwan.

## Methods

### Study population and study design

This was a retrospective, population-based, nested case–control study using clinical data retrieved from the Taiwan National Health Insurance Research Database (NHIRD). Patients with a diagnosis of HIV infection during the period from 1 January 2002 to 31 December 2016 were identified in the NHIRD. HIV infection is a notifiable disease in Taiwan and the cost of copayments for medical services for patients with HIV infection can be waived, and this can help to ensure the accuracy of the diagnosis of these patients.

### Data source

**Fig. 1:**
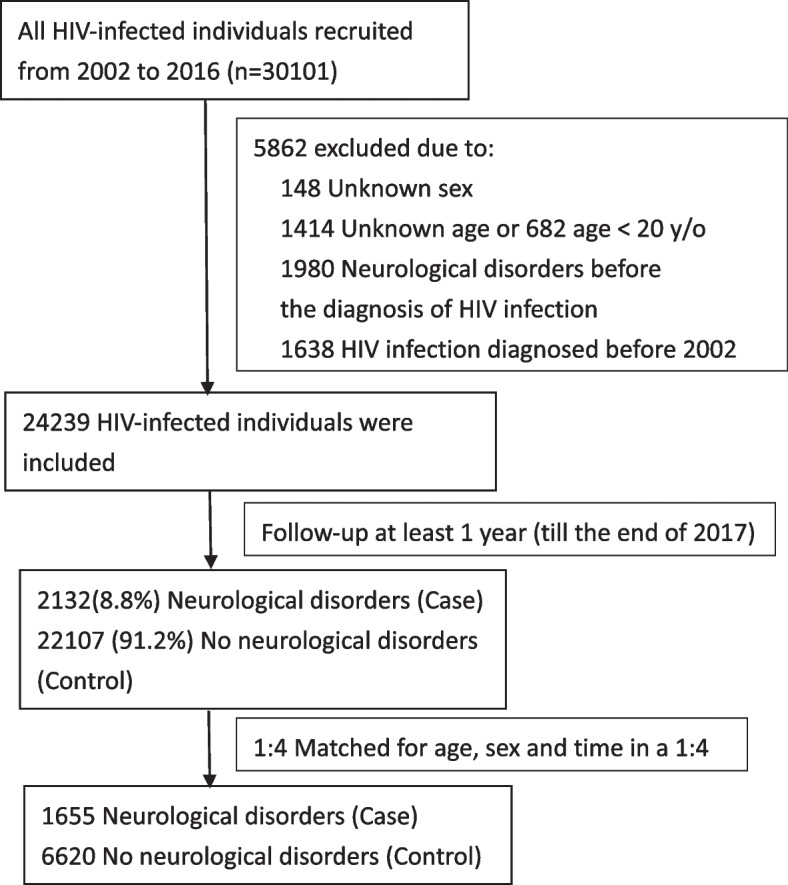
Flow chart of the HIV cohort for evaluating the risk of neurological disorders

By using the incidence of neurological disorders in HIV patients as the outcome variable, we excluded individuals with missing age or sex data and neurological disorders before the diagnosis of HIV infection. To estimate the effects of potential covariates on the risk of neurological disorders, a nested case–control study design with age, sex and time matching was applied in this study (Fig. 1). The primary outcome was the incidence of a first diagnosis of a neurological disorder after a diagnosis of HIV. Neurological disorders included neurocognitive disorders and central nervous system infections. The covariates were dyslipidemia, hepatitis C infection, substance use, alcoholism, traumatic brain injury, sleep apnea, sexually transmitted diseases, diabetes mellitus, psychiatric illnesses and HIV-associated opportunistic infections. These covariates were defined as the diagnoses recorded once or more during inpatient care or twice or more during ambulatory care within 1 year before the index date. Demographic profile (including sex, birth date, urbanization and monthly income), frequency of emergency department (ED) visits, and cART adherence were also extracted as covariates. The frequency of ED visits was analyzed because a previous study showed that ED visits were primarily driven by disease severity in people with HIV infection [25]. Adherence to cART was calculated as the proportion of days covered by dividing the number of days of ART coverage during the measurement period by the length of the measurement period [26]. Urbanization level was classified into urban, suburban and rural categories based on five aspects: population density, percentage of residents who were agricultural workers, the number of physicians per 100,000 people, percentage of residents with college or higher education, and percentage of residents aged 65 years or older [27].

Diagnoses in the NHIRD are coded based on International Classification of Diseases, Ninth Edition (ICD-9) and Tenth Edition (ICD-10) codes. ICD-9 codes were used between 2002 and 2014, and ICD-10 codes were used between 2015 and 2017. The ICD-9 and ICD-10 codes for the outcomes and covariates are provided in the Supplementary Table 1. The end of the observation period was defined as the occurrence of a neurological disorder, the end of 2017, or withdrawal from the National Health Insurance program.

This study was conducted after approval by the Institutional Review Board (IRB) of the National Cheng Kung University Hospital (B-EX-109–026). Since personal identification information is encrypted before releasing the data to researchers, informed consent was able to be waived from the IRB of the institute.

### Statistical analysis

Incidence rates were expressed per 1000 prospective person-years of observation from 2002 through 2017. Continuous variables were compared using the Student's t test, and categorical variables were compared using the chi-square test or Fisher's exact test. Variables significantly associated with the risk of neurological disorders in univariate conditional logistic regression analysis were then selected to construct the final multivariate logistic regression model. All statistical analyses were performed using SAS version 9.4 (SAS Institute, Cary, NC). A *p* value < 0.05 was considered to be statistically significant.

A nomogram is a two-dimensional diagram used to represent a mathematical function involving several predictors [28]. The variables significantly associated with the risk of neurological disorders in the multivariate logistic regression analysis were used to generate a nomogram.

## Results

### Demographic and clinical characteristics

**Fig. 2:**
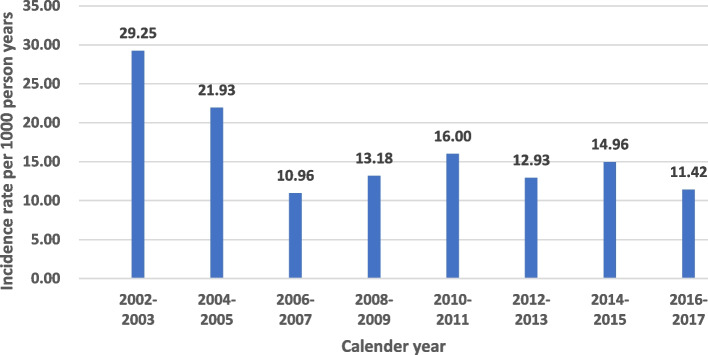
Incidence rate (per 1000 person-years) of neurological disorders among HIV-infected persons in Taiwan from 2002–2017

A total of 30,101 HIV-infected people were identified from 2002 to 2016, of whom 24,239 were used for further matching. A total of 2132 (8.8%) individuals were diagnosed with neurological disorders during the follow-up period. Of the 2132 HIV-infected people with neurological disorders, 87.27% were male and the mean age (± standard deviation) at diagnosis was 38.5 ± 14.7 years. About 65.45% of individuals received cART therapy. Among these 2132 individuals, 1168 (54.8%) individuals have central nervous system infections, and 997 (46.8%) individuals have neurocognitive disorders. Half of the neurological disorders were identified before the initiation of cART. The proportion of central nervous system infections and neurocognitive disorders were quite similar before and after starting cART. The overall incidence of neurological disorders was 13.67 per 1000 person-years (Fig. 2). The incidence of central nervous system infections was 7.49 per 1000 person-years, and the incidence of neurocognitive disorders was 6.40 per 1000 person-years. The median time from the index date to a diagnosis of a neurological disorder was 3.6 years. The individuals with (case group) and without (control group) a neurological disorder were then matched by age, sex and time. The cases and controls were selected at a 1:4 ratio (Fig. 1). Table 1 shows the demographic and clinical characteristics of the case (*n* = 1655) and control (*n* = 6620) groups.

**Table 1:** Demographic and clinical characteristics of the neurological disorders and control subjects used to identify possible risk factors in HIV-infected persons

	Control subjects (*N* = 6620)	Patients with neurological disorders (*N* = 1655)
Variables	n (%)	n (%)
**Duration of HIV infection (years)**		
Median (IQR)	3.62 (1.23, 6.69)	3.63 (1.21, 6.70)
**Sex**		
Male	6472 (97.76)	1618 (97.76)
Female	148 (2.24)	37 (2.24)
**Age (years)**		
Mean ± SD	32.9 ± 7.2	33.0 ± 7.2
**Comorbidities (2 year before index date)**		
Dyslipidemia	134 (2.02)	33 (1.99)
Hepatitis C infection	180 (2.72)	88 (5.32)
Substance use	418 (6.31)	195 (11.78)
Alcoholism	35 (0.53)	32 (1.93)
Traumatic brain injury	59(0.89)	34 (2.05)
Sleep apnea	11 (0.17)	4 (0.24)
Sexually transmitted disease	1319 (19.92)	268 (16.19)
Diabetes mellitus	86 (1.30)	32 (1.93)
Psychiatric illness	784 (11.84)	316 (19.09)
HIV-associated OIs	519 (7.84)	170 (10.27)
**Frequency of ED visits**		
None	3404 (51.42)	510 (30.82)
1–2 times annually	2671 (40.35)	794 (47.98)
3–5 times annually	473 (7.15)	279 (16.86)
>5 times annually	72 (1.09)	72 (4.35)
**cART adherence**		
<90% PDC	6514 (98.40)	1640 (99.09)
≥ 90% PDC	106 (1.60)	15 (0.91)
**Urbanization**		
Urban	2027 (30.62)	395 (23.87)
Suburban	1461 (22.07)	306 (18.49)
Rural	3132 (47.31)	954 (57.64)
**Monthly income**		
NT$≤ 15,840	2958 (44.68)	879 (53.11)
NT$ 15,841–25,000	1888 (28.52)	513 (31.00)
≥ NT$ 25,001	1774 (26.80)	263 (15.89)

*HIV* Human immunodeficiency virus, *IQR* Interquartile range, *SD* Standard deviation, *OIs* Opportunistic infections, *ED* Emergency department, *PDC* Proportion of days covered, *NT$* New Taiwan dollar

### Factors associated with neurological disorders in the HIV-infected persons

Risk factors included in conditional logistic regression analysis were age at HIV diagnosis, dyslipidemia, hepatitis C infection, substance use, alcoholism, traumatic brain injury, sleep apnea, sexually transmitted diseases, diabetes mellitus, psychiatric illnesses, HIV-associated opportunistic infections, frequency of ED visits, cART adherence, urbanization level and monthly income. Odds ratios, adjusted odds ratios and their corresponding upper and lower 95% confidence intervals are presented in Table 2. In the univariate analysis, older age, hepatitis C infection, substance use, alcoholism, traumatic brain injury, sexually transmitted diseases, psychiatric illnesses, HIV-associated opportunistic infections, frequency of ED visits, cART adherence, urbanization and monthly income were associated with neurological disorders. Dyslipidemia, sleep apnea and diabetes were not associated with neurological disorders. In the multivariate analysis, hepatitis C infection, alcoholism and sexually transmitted diseases were no longer significant. Due to concerns about confounding by age, we then performed subgroup analyses of only younger subjects (arbitrarily defined as less than 40 years of age) and only older subjects (40 years or older). The results are shown in Table 3.

**Table 2:** Crude and adjusted odds ratios of neurological disorders in HIV-infected persons

Variables	Crude OR (95% CI)	*p*-value	^a^Adjusted OR (95% CI)	*p*-value
**Age (years)**	1.11 (1.05–1.17)	<0.001*	1.10 (1.04–1.16)	<0.001*
**Comorbidities (2 year before index date)**				
Dyslipidemia	0.98 (0.66–1.46)	0.936		
Hepatitis C infection	2.01 (1.55–2.61)	< 0.001*	1.27 (0.95–1.69)	0.103
Substance use	2.06 (1.71–2.48)	<0.001*	1.35 (1.10–1.67)	0.005*
Alcoholism	3.71 (2.29–6.02)	< 0.001*	1.26 (0.74–2.16)	0.398
Traumatic brain injury	2.36 (1.54–3.63)	< 0.001*	1.69 (1.06–2.70)	0.026*
Sleep apnea	1.46 (0.46–4.57)	0.521		
Sexually transmitted disease	0.77 (0.67–0.89)	0.001*	0.88 (0.76–1.03)	0.117
Diabetes mellitus	1.50 (1.00–2.26)	0.053	1.12 (0.72–1.73)	0.621
Psychiatric illness	1.76 (1.52–2.03)	<0.001*	1.34 (1.15–1.57)	<0.001*
HIV-associated OIs	1.35 (1.13–1.63)	0.001*	1.25 (1.03–1.52)	0.026*
**Frequency of ED visits**				
None			Ref.	
1–2 times annually	2.18 (1.92–2.48)	<0.001*	2.12 (1.86–2.41)	<0.001*
3–5 times annually	4.44 (3.69–5.34)	<0.001*	4.03 (3.34–4.87)	<0.001*
>5 times annually	7.67 (5.38–10.92)	<0.001*	5.90 (4.09–8.52)	<0.001*
**cART adherence (in observation period)**				
<90%	Ref.		Ref.	
≥90%	0.48 (0.26–0.88)	0.018*	0.37 (0.20–0.71)	0.003*
**Urbanization**				
Urban	Ref.		Ref.	
Suburban	1.07 (0.91–1.26)	0.415	0.95 (0.80–1.12)	0.524
Rural	1.61 (1.41–1.84)	<0.001*	1.31 (1.13–1.51)	<0.001*
**Monthly income**				
NT$ ≤ 15,840	Ref.		Ref.	
NT$ 15,841–25,000	0.91 (0.80–1.03)	0.117	0.98 (0.86–1.12)	0.807
≥ NT$ 25,001	0.48 (0.41–0.56)	<0.001*	0.61 (0.52–0.72)	<0.001*

*OR* Odds ratio, *CI* Confidence interval, *ED* Emergency department, *cART* Combination antiretroviral therapy, *NT$* New Taiwan dollar

^*^p-value ≤ 0.05

^a^Adjusted for age, hepatitis C infection, substance use, alcoholism, traumatic brain injury, sexually transmitted disease, psychiatric illness, HIV-associated OIs, frequency of ED visit, cART adherence, urbanization and monthly income

**Table 3:** Adjusted odds ratios of neurological disorders in HIV-infected persons, stratification by age

Variables	**20 ≤ Age < 40**		**Age ≥ 40**	
	Adjusted OR (95% CI)	*p*-value	Adjusted OR (95% CI)	*p-*value
**Comorbidities (2 year before index date)**				
HCV infection	1.34 (0.95–1.88)	0.094	1.12 (0.64–1.97)	0.696
Substance use	1.45 (1.13–1.86)	0.003*	1.01 (0.66–1.54)	0.963
Alcoholism	1.23 (0.66–2.29)	0.524	1.45 (0.48–4.40)	0.516
Traumatic brain injury	1.42 (0.83–2.42)	0.200	2.76 (0.98–7.78)	0.055
Sexually transmitted disease	0.87 (0.73–1.03)	0.097	0.93 (0.60–1.45)	0.760
Diabetes mellitus	0.98 (0.52–1.84)	0.952	1.24 (0.64–2.40)	0.522
Psychiatric illness	1.23 (1.03–1.48)	0.024*	1.78 (1.27–2.51)	0.001*
HIV-associated OIs	1.37 (1.10–1.72)	0.005*	0.95 (0.62–1.45)	0.806
**Frequency of ED visits**				
None	Ref.		Ref.	
1–2 times annually	2.18 (1.88–2.53)	<0.001*	1.89 (1.39–2.56)	<0.001*
3–5 times annually	4.22 (3.41–5.22)	<0.001*	3.19 (2.06–4.93)	<0.001*
> 5 times annually	5.36 (3.58–8.01)	<0.001*	14.20 (4.74–42.54)	<0.001*
**cART adherence (in observation period)**				
<90%	Ref.		Ref.	
≥90%	0.41 (0.21–0.81)	0.011*	0.16 (0.02–1.34)	0.091
**Urbanization**				
Urban	Ref.		Ref.	
Suburban	0.97 (0.80–1.17)	0.737	0.98 (0.64–1.50)	0.930
Rural	1.29 (1.10–1.52)	0.002*	1.54 (1.07–2.23)	0.021*
**Monthly income**				
NT$≤15,840	Ref.		Ref.	
NT$ 15,841–25,000	0.95 (0.82–1.10)	0.481	1.12 (0.83–1.52)	0.467
≥ NT$ 25,001	0.60 (0.50–0.73)	<0.001*	0.67 (0.45–0.99)	0.044*

*OR* Odds ratio, *CI* Confidence interval, *ED* Emergency department, *cART* Combination antiretroviral therapy, *NT$* New Taiwan dollar

^*^p-value ≤ 0.05

### Nomogram

**Fig. 3:**
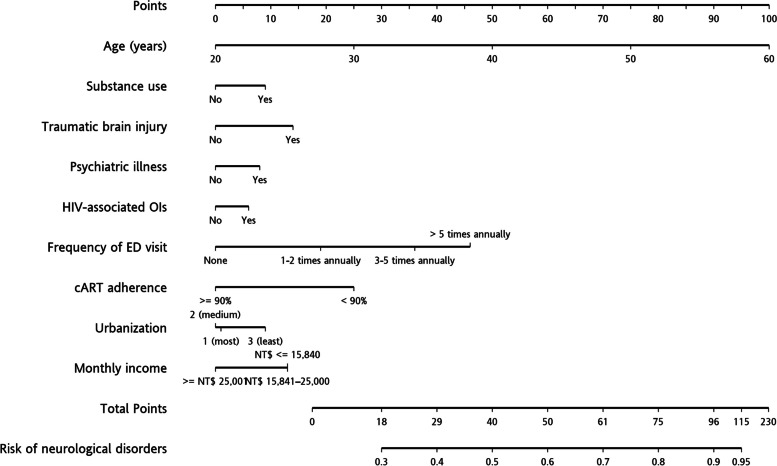
Nomogram for predicting the development of neurological disorders in HIV-infected persons

According to the multivariate analysis results, a nomogram was generated to estimate the risk of developing a neurological disorder as shown in Fig. 3. By summing the risk score for each factor as shown in the nomogram, the risk of developing a neurological disorder for each individual can be assessed.

## Discussion

In this retrospective nested case–control study, we found several risk factors for neurological disorders in HIV-infected people and then developed a simple risk scoring system to identify those at risk. To the best of our knowledge, this scoring system is the first to be specifically designed for identifying neurological disorders in people infected with HIV. Several clinical factors and comorbidities have been reported to be associated with neurological disorders in HIV-infected people, including the frequency of ED visits [29], cART adherence [30, 31], advanced HIV disease [17], duration of HIV infection [20, 21], and older age [23]. Comorbidities including obesity, diabetes [22], hepatitis C infection [23], alcohol use, substance abuse, traumatic brain injury, sleep disorders and psychiatric illnesses [24] have also been associated with neurological disorders in HIV-infected people. The large number of factors which can contribute to the development of neurological disorders in this population makes it more complex to predict. Through the proposed nomogram with some basic clinical information, clinicians can identify those at risk and initiate further screening for comorbidities, drug compliance education, or even cognitive function evaluations. This nomogram may serve as a screening tool for identifying risk populations.

Educational attainment [32], tobacco use [33], and cART regimen [34, 35] can also influence neurocognitive function. Since educational attainment is closely related to the level of income [36, 37] and monthly income could be extracted from the NHIRD, we used monthly income as a covariate instead of educational attainment as data on educational attainment are not available in the NHIRD. However, more research is needed to evaluate whether adding more parameters (clinical factors and/or biomarkers) could better predict the development of neurological complications in HIV-infected people.

The incidence of neurological disorders in HIV-infected persons was stable from 2006 to 2017 (13.67 per 1000 person-years) even though early treatment and even a "treat all" policy was applied during this period and more tolerable modern cART was used. This finding is consistent with previous studies in which neurological complications were still prevalent in HIV-infected persons due to it being neuroinvasive, neurotropic and neurovirulent [38, 39]. Thus, neurological manifestations are an important concern among people with HIV infection.

In the subgroup analyses of only younger subjects and only older subjects, substance use was significantly associated with neurological disorders in the younger subjects(adjusted HR = 1.45, *p* = 0.003), but not in the older subjects(adjusted HR = 1.01, *p* = 0.963). This may be because substance use is typically higher in adolescents and young adults, and the neurological complications of substance use can occur in both acute and early HIV infection [40]. This should raise awareness of neurological disorders in young HIV-infected people with substance use disorders.

The key strength of this study is the application of a nationwide database to identify predictors of neurological disorders. The high coverage, easy accessibility, and low copayments result in high adherence of beneficiaries to the National Health Insurance program, which minimizes potential selection and information biases.

Some limitations should also be addressed. First, some risk factors for neurological disorders such as low CD4 cell count, high blood viral load, low educational attainment, tobacco use and cART regimen are not included in the NHIRD and could not be incorporated into the scoring system. Both CD4 cell count and blood viral load are important predictors of outcomes in HIV-infected persons [17, 41]. In addition, we used HIV-associated opportunistic infections as a proxy for advanced HIV status. Second, the diagnosis of neurological disorders and comorbidities depended on claims data from the NHIRD, and physicians who cared for these patients were not neurologists, which may have led to underestimation of the proportion of neurological disorders. Third, cART adherence was calculated by the proportion of days covered, and the actual adherence rate may have been lower, especially in those with neurological disorders [42, 43].

In conclusion, neurological disorders are still prevalent in HIV-infected persons. To efficiently identify those at risk, we established a nomogram with nine risk factors. This nomogram could prompt clinicians to initiate further evaluations and management of neurological disorders.

### Supplementary Information

**Additional file 1: Supplementary Table 1.** ICD-9 and ICD 10 codes used for neurological disorders and covariates.

## Data Availability

The de-linked datasets used and/or analysed during the current study are available from the corresponding author on reasonable request. The data are not publicly available because the use of the National Health Insurance Research Database is limited to research purposes only.

## Abbreviations

cART: Combination antiretroviral therapy
ED: Emergency department
HIV: Human immunodeficiency virus
ICD-9/10: International Classification of Diseases, Ninth/Tenth Edition
NHIRD: National Health Insurance Research Database
PLWH: People living with HIV
