# Supplementary material for: Prediction of incidence of neurological disorders in HIV-infected persons in Taiwan: a nested case–control study
Source: BMC Infect Dis. 2023 Nov 4;23:759. doi: 10.1186/s12879-023-08761-4 (PMC10625280; doi:10.1186/s12879-023-08761-4)
Supplement: Supplementary file 1 — Additional file 1: Supplementary Table 1. ICD-9 and ICD 10 codes used for neurological disorders and covariates. [file 12879_2023_8761_MOESM1_ESM.pdf]

Supplementary Table 1. ICD-9 and ICD-10 codes used for neurological disorders and covariates

Description	ICD-9 codes	ICD-10 codes
HIV infection	042	B20
Cognitive disorders	290, 293, 294, 331, 332.1, 333.0, 345., 348.3, 780.3x	F03, F04, F05, F0150, F0151, F06x, F06xx, F0280, F0281, G138, G21x, G2111, G2119, G23x, G30x, G311, G312, G318x, G319, G20, G903, G40xx, G40xxx, G934x, R569, R5600, R5601
CNS infection	013, 036, 045, 046, 047, 048, 049, 052.0, 053.0, 053.1, 054.3, 054.72, 055.0, 056, 062, 063, 064, 098.82, 100.81, 139.0, 320, 321, 322, 323, 324, 326	A170, A171, A178, A179, A2781, A390, A3981, A5481, A80, A81xx, A83, A84, A85, A86, A87, A89, B0111, B010, B0112, B020, B021, B0229, B0221-B0224, B0229, B004, B1001, B1009, B003, B0082, B050, B051, B0600, B0601, B0602, B451, B060x, , B941, G00, G01, G02, G03, G04, G05, G06, G07, G08, G09, G373, G06, G92
Dyslipidemia	272.0-272.4	E780-E785
Hepatitis C infection	070.41, 070.44, 070.51, 070.54	B1719, B1711, B182, B1920, B1921
Substance use	292, 304, 305, E935.0	F11, F12, F13, F14, F15, F16, F17, F18, F19
Alcoholism	291, 303, 305.0x, 357.5, V11.3	F10, G621
Traumatic brain injury	800, 801, 850, 851	G913, S064x0A, S060, S065, S066, S063, S069x9A
Sleep apnea	780.5	G473
Sexually transmitted disease	091, 092, 093, 094, 095, 096, 097, 098, 099	A510, A5209, A521, A522, A523, A527, A528, A529, A530, A539, A54, A55, A56, A57, A58, A638, A64
Diabetes mellitus	250	E10xx, E10xxx, E108, E109, E11
Psychiatric illness	295, 296, 297, 298, 299, 300, 301, 306, 307.1, 307.5, 308, 309, 310, 311, 312, 313, 314	F0789, F079, F09, F20, F21, F22, F23, F24, F25, F28, F3xx, F3xxx, F4xx, F4xxx, F42, F502, F509, F60, F63, F84, F99, F9xx
HIV-associated opportunistic infections	002, 003, 007.4, 010, 012, 014, 015, 016, 017, 018, 031, 046.3, 054, 078.5, 112, 114, 115, 117.5, 130, 136.3, 137, 176, 180, 200, 481, 482, 483, 484.1, 485, 486	A01, A02, A072, A073, A15, A18, A19, A31, A812, B001, B005, B007, B008, B250, B258, B259, B37, B38, B39, B45, B46, B58, B59, B90, C53, C830, C837, J13, J14, J15, J160, J168, J18
